# Differential Expression of Cell Wall Remodeling Genes Is Part of the Dynamic Phase-Specific Transcriptional Program of Conidial Germination of *Trichoderma asperelloides*

**DOI:** 10.3390/jof8080854

**Published:** 2022-08-15

**Authors:** Maggie Gortikov, Elizabeta Yakubovich, Zheng Wang, Francesc López-Giráldez, Yujia Tu, Jeffrey P. Townsend, Oded Yarden

**Affiliations:** 1 Department of Plant Pathology and Microbiology, The RH Smith Faculty of Agriculture, Food and Environment, The Hebrew University of Jerusalem, Rehovot 7610001, Israel; 2 Department of Biostatistics, Yale School of Public Health, New Haven, CT 06511, USA; 3 Yale Center for Genomic Analysis, New Haven, CT 06511, USA; 4 Department of Mathematics and Computer Science, University of Strasbourg, 67081 Strasbourg, France

**Keywords:** conidial germination, mycoparasite, cell wall remodeling, chitinase, glucanase, chitin synthase, glucan elongase, Congo Red

## Abstract

The nature of saprophytic and mycoparasitic hyphal growth of *Trichoderma* spp. has been studied extensively, yet its initiation via conidial germination in this genus is less well understood. Using near-synchronous germinating cultures of *Trichoderma asperelloides*, we followed the morphological progression from dormant conidia to initial polar growth to germling formation and to evidence for first branching. We found that the stage-specific transcriptional profile of *T. asperelloides* is one of the most dynamic described to date: transcript abundance of over 5000 genes—comprising approximately half of the annotated genome—was unremittingly reduced in the transition from dormancy to polar growth. Conversely, after the onset of germination, the transcript abundance of approximately a quarter of the genome was unremittingly elevated during the transition from elongation to initial branching. These changes are a testimony to the substantial developmental events that accompany germination. Bayesian network analysis identified several chitinase- and glucanase-encoding genes as active transcriptional hubs during germination. Furthermore, the expression of specific members of the chitin synthase and glucan elongase families was significantly increased during germination in the presence of *Rhizoctonia solani*—a known host of the mycoparasite—indicating that host recognition can occur during the early stages of mycoparasite development.

## 1. Introduction

Spores are reproductive units of prokaryotic and eukaryotic organisms, including algae and protozoa, lower vascular plants, and even a subset of animals, produced to propagate their genetic material [1]. Many members of the fungal kingdom can produce sexual and/or asexual spores in an astonishing variety of shapes, sizes, and other features adapted to specific lifestyles and the requirements for dispersal or persistence in different environments [2]. The fungal asexual spore—or conidium—is a nonmotile, walled, predominantly-haploid cell, usually generated by mitosis from a parent cell. Asexual spore production is a critical stage in the life cycle of fungi, serving as the primary means for dispersion, infection, and survival under adverse conditions [1,3].

Conidial germination is composed of a sequence of events that convert the resting propagule into a rapidly growing germ tube from which the mycelium will be formed by subsequent elongation and branching [4]. It differs from later hyphal growth in the marked dependence of each conidium on its stored reserves for metabolism. Physiologically, it is characterized by transformation of a conidium from a stage of low to one of high metabolic activity [5]. Despite its low metabolic activity, the dormant conidium has been shown to be transcriptionally active [6]. The morphological progression of conidial germination has been divided into four main stages, whose intervening transitions between have been associated with metabolic shifts: (1) dormant conidia, (2) evidence for polar growth, (3) doubling of the long axis of the conidium, and (4) evidence for first branching [7].

Once germination is triggered, numerous metabolic activities, including respiration, RNA and protein synthesis, breakdown of stored carbohydrates, and remodeling of the cell wall, are enhanced [1,8]. These activities are accompanied by an increase in intracellular osmotic pressure, followed by water uptake, causing the conidium to swell. This isotropic swelling is the first morphological change that can be observed during conidial germination and is concomitant with the initiation or intensification of numerous metabolic activities. The next developmental phase involves establishment of polarity and the formation of a germ tube. The production of a germ tube that is as broad as it is long has been used to define the transition point between this phase and the following steps of vegetative growth: hyphal elongation and branching [4,9].

Conidial germination has been studied in a variety of ascomycete filamentous fungi, including *Aspergillus niger* [10], *A. fumigatus* [11], *Fusarium gramineraum* [12], and *Neurospora crassa* [7,13]. These filamentous fungi all undergo similar morphological processes during germination. However, the conditions under which every fungus germinates are varied phenotypically, temporally, and genetically. Furthermore, at least some features of germination can be affected by the environmental conditions that were prevalent during conidiogenesis [6].

*Trichoderma* spp. are filamentous–ascomycete fungi of the family Hypocreaceae [14,15]. Fungi of this genus are ubiquitous, highly adaptable, and are found worldwide. *Trichoderma* spp. are among the most prevalent culturable fungi and are frequently isolated from forests or agricultural soils. They have also been found in the rhizosphere, on dead and rotting wood, and in association with other fungi [16]. Members of the genus *Trichoderma* have also been found in association with marine sponges and other aqueous environments, in air and settled dust, as plant endophytes, and as organisms opportunistically infecting humans [17,18,19,20,21].

Lifestyles of *Trichoderma* spp. are diverse and versatile, and, while saprotrophy, necrotrophy, and biotrophy have been the major traditional nutritional modes that have been referred to, evidence for hemibiotrophy has also been documented [17,22,23]. As saprotrophs, some *Trichoderma* species contribute to the degradation of plant debris, wood, and bark, and have been used for industrial production of cellulases and hemicellulases [24]. As necrotrophs, many *Trichoderma* species live in parasitic relationships, feeding on a living host, usually as mycoparasites of other fungi. To this end, species such as *T. asperellum*, *T. atroviride*, *T. virens*, and *T. harzianum* have been used as biological control agents of plant pathogens, such as *Alternaria alternata*, *Botrytis cinerea*, *Sclerotinia sclerotiorum*, *Rhizoctonia solani,* and others [25].

The mycoparasitic abilities of *Trichoderma* are complex, involving the detection of the target host through chemotropism, lysis of the host’s cell wall, hyphal coiling around the host, penetration by appressorial formation, production of cell-wall-degrading enzymes, and consumption of the contents of the host cell [14,26]. These highly developed mycoparasitic properties of *Trichoderma* spp. have been harnessed as a biological control agent in agriculture. In addition, their capacity to produce large quantities of lytic enzymes and antimicrobial secondary metabolites has made the genus industrially important and well-studied. Over 50 Trichoderma-based biofungicide formulations have been developed, and the genus now accounts for 60% of the fungi used as biological control agents globally [17,27,28].

While much attention has been paid to the processes of host detection and hyphal coiling, not much is known concerning the interaction between *Trichoderma* with its potential hosts in their early pre-parasitic association. Specifically, information regarding the dynamic process of germination of the mycoparasite in the presence of the host is lacking. The initial point of mycoparasite contact occurs at the cell wall of the host, which modulates fungal interactions [23]. The cell wall is an essential component of the fungal cell, providing strength, structure, and protection against environmental stress [29,30]. The conidial cell wall is composed of polysaccharides, proteins, lipids, and pigments. Predominant components include glucans, chitin, chitosan, and glycosylated proteins. Glucan and chitin are synthesized by plasma-membrane-associated glucan synthases and chitin synthases, respectively, while cell wall glycoproteins are synthesized by ER-associated ribosomes. In concert with the biosynthetic protein machinery, an array of glycosyl hydrolases participate in shaping and remodeling the fungal cell wall [31].

During the transition from isotropic growth to polarized growth, and then to germ- tube formation and elongation, the conidial cell wall undergoes significant modifications [9,32]. To perform these modifications, most fungal genomes encode multiple chitin synthases. Some chitin synthases have been demonstrated to function in specific cell types or during restricted phases of the cell cycle [32]. This multiplicity and specificity is exemplified by the eight chitin synthases of *T*. *atroviride*, which have been identified and demonstrated to be differentially expressed during germination and hyphal elongation, as well as differentially affected by the intercalating dye Congo Red [33]. The presence of multiple glycosyl hydrolases—many of them conserved—is also common in fungal species. Members of glycosyl hydrolase family 72 are among the best-characterized cell wall remodeling enzymes. These GPI-anchored enzymes execute an essential role in cell wall biogenesis; for example, β-(1,3)-glucanosyltranferases (GELs) are a major functional component of β-(1,3)-glucan processing [34,35,36,37], and their involvement in conidial germination has also designated some of them as potential targets for antifungals [38].

One of the intensively studied *Trichoderma* spp. strains is *T. asperelloides* T203. This strain was formerly identified as *T. asperellum*, and prior to that as *T. harzianum*, and is a cryptic sister species to *T. asperellum* [39]. These two species belong to the section Trichoderma, which contains several other prominent mycoparasitic species, such as *T. atroviride*, *T. gamsii*, and *T. hamatum*. The host range of *T. asperelloides* T203, along with traits involved in host recognition and some of the interactions between this mycoparasite and its hosts, has been analyzed [40,41]. These studies were largely devoted to characterizing the activity of enzymes involved in cell wall degradation/remodeling. Importantly, the potential to use *T. asperelloides* T203 for biocontrol has been extensively tested in the greenhouse, as well as in the field [42].

In this study, we analyzed the transcriptional profile of the recently sequenced *T. asperelloides* strain T203 [43] (whose genetic attributes are summarized at https://mycocosm.jgi.doe.gov/vista_embed/?organism=Triasper1 (accessed on 5 July 2022). Moreover, we assessed the degree of dynamism of transcript abundance during germination of this species. Examining the changes occurring in genes encoding cell wall remodeling proteins, we elucidated how the transcription of at least some members of the chitin synthase and glucan elongase families are affected by the presence of *R. solani*, a potential host of the mycoparasite.

## 2. Materials and Methods

### 2.1. Strains and Culturing Conditions 

*Trichoderma asperelloides* strain T203 [43] and *Rhizoctonia solani* TP6 [44] were cultured on Potato Dextrose agar or broth (PDA or PDB, respectively. Difco Laboratories, Detroit, MI, USA) at 28 °C. Conidia of *T. asperelloides* strain T203 were harvested from 5–7-day-old cultures. Conidia were harvested from three 90 mm diameter Petri dishes by adding 5 mL of cold sterile double deionized water (DDW) to each dish. Conidia were released by brief shaking with glass beads and collected by filtration through a gauze pad layered over a funnel. The conidial suspension was centrifuged at 4000 rpm for 5 min, and the conidial pellet was washed twice with 30 mL cold DDW. Conidial suspensions were incubated overnight at 4 °C and then conidial density was calculated using a hemocytometer and adjusted to a concentration of 5 × 10^6^ conidia/mL. To facilitate the harvesting of conidia and germlings cultured on PDA, presterilized 40-µm pore cellulose membranes (Life Technologies, Carlsbad, CA, USA) were placed over the solid medium prior to spreading the inoculum. Plates were inoculated by spreading 50 μL of the adjusted conidial suspension and incubated at 28 °C for the required periods. For growth in PDB, conidial suspensions were first rehydrated as described above. The PDB medium (50 mL) was inoculated to a final concentration of 5 × 10^6^ conidia/mL. Cultures were incubated at 28 °C, at 160 rpm, for the required time. When necessary, Congo Red (Sigma, St. Louis, MO, USA),) was added to PDB medium at the desired concentration prior to autoclaving. Cultures were sampled every hour and examined by light microscopy (EVOS FL Auto Cell Imaging System—Life Technologies, Carlsbad, CA, USA) for timing fresh conidia, polar growth, doubling of the long axis, and first hyphal branching. The timing experiment was repeated three times.

To analyze germination in the presence of a potential host of the mycoparasite, *R. solani* was first cultured on PDA for 5–7 days. *R. solani* plates were overlaid with sterile cellulose membranes, inoculated with 50 μL of adjusted conidial suspensions of *T. asperelloides*, and incubated at 28 °C. Cultures were sampled after a range of intervals (within 0–48 h) and examined by light microscopy.

### 2.2. Isolation of Nucleic Acids, RT-PCR, and RNASeq

RNA was isolated from *T. asperelloides* cultures after freezing in liquid nitrogen and grinding to a powder using a mortar and pestle, using the TRIzol reagent and the 1- Bromo-3-chloropropane (BCP, Sigma Aldrich) method, as previously described [45]. RNA samples were further purified with the RNA Clean and ConcentratorTM-5 kit (Zymo Research, Irvine, CA, USA) and treated with DNase (RNase-Free DNase Set, Qiagen, Hilden, Germany) according to the manufacturer’s instructions. RNA yield and quality were assessed using the Synergy™ HTX Multi-Mode Reader (Biotek, Winooski, VT, USA) and verified using gel electrophoresis.

For RT-PCR, RNA was reverse transcribed using the High-Capacity cDNA Reverse Transcription kit (Applied Biosystems, Waltham, MA, USA). Transcript abundance was determined using a Step One Plus RealTime PCR (Applied Biosystems, Waltham, MA, USA) apparatus using gene-specific primers (Table 1) under standard reaction conditions as previously described [46]. To estimate the relative amounts of transcripts in each sample, we calculated 2^−^^ΔΔCT^, where ΔΔCT is the ΔCT of the test sample and −ΔCT of the calibrator sample, and ΔCT is the difference between the cycle threshold (CT) of the gene of interest and the reference gene. Translation elongation factor 1α (tef1α) was used as a reference gene.

To prepare cDNA samples for RNAseq, mRNA was purified from approximately 200 ng of total RNA with oligo-dT beads and sheared by incubation at 94 °C in the presence of Mg (Roche Kapa mRNA Hyper Prep Catalog # KR1352). Further, first-strand cDNA-tailing was performed with dUTP to generate strand-specific sequencing libraries. Indexed libraries were quantified by qRT-PCR using a commercially available kit (Roche KAPA Biosystems Cat # KK4854). The quality of cDNA samples was verified with a bioanalyzer (Agilent Technologies 2100; Agilent Technologies, Santa Clara, CA, USA). In total, 12 libraries were constructed from the four developmental stages, each with 3 biological replicates. Ten libraries passed initial quality control and were sequenced. Basic statistics, such as total reads sequenced and mapping rates, are summarized in Table S1. The cDNA samples were sequenced at the Yale Center for Genomics Analysis (YCGA). The libraries underwent 101-bp single-end sequencing using an Illumina HiSeq 2500 in Rapid Run Mode according to Illumina protocols. Adapter sequences, empty reads, and low-quality sequences were removed. Reads were trimmed using fastp v0.21.0 [47] using default parameters. Trimmed reads were aligned to the *Trichoderma asperelloides* T203 v1.0 genome from GenBank [43] using HISAT2 v2.1, indicating that reads correspond to the reverse complement of the transcripts and reporting alignments tailored for transcript assemblers. Alignments with a quality score below 20 were excluded from further analysis. Reads were counted for each gene with StringTie v1.3.3 and the Python script prepDE.py provided in the package. StringTie was limited to report reads that matched the reference annotation. Transcriptomics data and experiment details were made available (GSE207066) at the GEO database (https://www.ncbi.nlm.nih.gov/geo/ (accessed on 5 July 2022). Genome-wide gene expression was profiled across all four sampled conidial germination stages using Deseq2 [48] LOX v1.6 [49]. Differentially expressed genes (DEGs), including downregulated and upregulated genes, were identified between stages based on the LOX measurement, and a gene expression difference with a LOXP value < 0.05 was deemed statistically significant. Functional enrichment analysis for DEGs significantly up- or downregulated during the conidial germination was based on the GO term annotation of the genome *T. asperelloides* using *T. asperellum* as a key reference. Significant enrichment of GO terms was identified using Fisher’s Exact Test, adjusted with the Benjamini–Hochberg procedure [50] to *p* < 0.05.

### 2.3. Bayesian Network Prediction for Functional Genes Groups during Conidial Germination

Functional annotation was based on the JGI fungal genome database. Additional functional annotations were obtained by comparing *T. asperelloides* with the extensively studied and annotated genome of *T. asperellum*. *T. asperelloides* homologs were identified using OrthoLoger [51]. Bayesian gene networks were generated to reveal gene interactions, associations, and regulatory modules involved in conidial germination in *T. asperelloides*. Biological networks were modeled for selected genes of interest using the Bayesian Network Web Server [52]. Input files consisted of fold changes between adjacent sample points across the four time points of the experiment. Fold changes between stages were calculated from LOX measurements of conidial germination on PDA using change in expression over the minimum expression between the stages, as previously described [53,54]. Global structure-learning settings were retained at default settings. The models depicted were the 50% majority consensus of 100 models (selection threshold set to 0.5; the 100 highest-scoring networks were averaged), without imposing any structural constraints. Edges with posterior probability higher than 0.5, based on the consensus of 100 models, were present in the figures.

## 3. Results

### 3.1. Phenotypic Characteristics of Germinating T. asperelloides Strain T203

**Figure 1:**
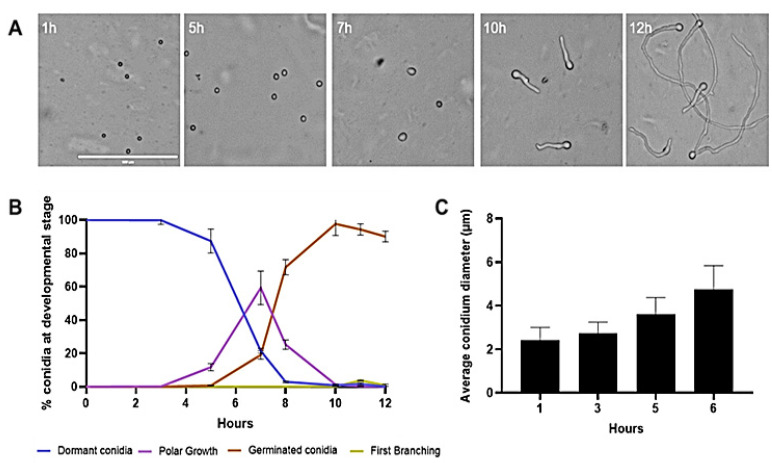
Phenotypic characterization of conidia germinating on PDA. (**A**) Conidia germinated in PDA were imaged at 1, 5, 7, 10, and 12 h; scale bar: 100 µm for all images). (**B**) The degree of synchronous progression of germination was monitored and cells were assigned as either dormant conidia, polar growth, doubling of long axis, or first hyphal branching. (**C**) Average diameters of conidia were assessed during the early stages of germination; whiskers indicate the standard deviation of the mean for the three biological replicates.

Germination of rehydrated conidia of *T. asperelloides* on PDA overlaid with a cellophane membrane was monitored over time. Conidial swelling—characterized by an isotropic increase in size—continued for five to seven hours in a near-synchronous manner (Figure 1A,B). During the course of this transition between resting and fully swollen conidia, the average conidium diameter increased from 2.4 µm to 4.75 µm (Figure 1C). Once fully swollen, germ tubes emerged from the conidia. By nine to twelve hours, most conidia (90%) produced hyphae that exceeded the diameter of the conidium in length. The first branching of the hyphae was observed after twelve hours.

### 3.2. Changes in Gene Expression during Germination of T. asperelloides Strain T203

**Figure 2:**
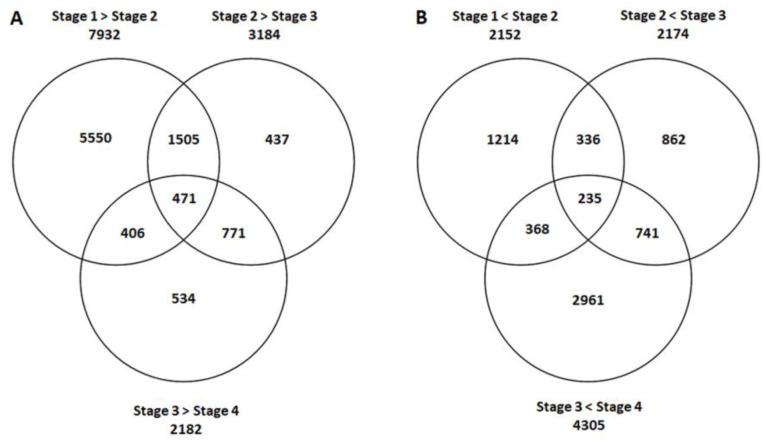
Summary of the number of genes significantly (**A**) down- or (**B**) up- regulated across stages of germination (Stage 1→Stage 2: dormant conidia to polar growth; Stage 2→Stage 3: polar growth to germinated conidia; and Stage 3→Stage 4: germinated conidia to first branching).

We used RNA sequencing to determine the extent of changes in transcript abundance during the four phases of conidial germination. Among the levels of expression of a total of 11,166 genes that were measured (Figure 2; Table S2), 7932 genes exhibited significant downregulation (*p* < 0.05; exhibiting lower transcript abundance) and 2152 genes were significantly “upregulated” (exhibiting higher transcript abundance) from stage one to stage two (dormant conidia to polar growth). From stage two to stage three (polar growth to germinated conidia), 3184 genes exhibited significant downregulation and 2174 genes exhibited significant upregulation. From stage three to stage four (germinated conidia to first branching), 2182 genes exhibited significant downregulation and 4305 genes exhibited significant upregulation.

Among the 2152 genes whose expression was upregulated from dormant conidia to commencement of polar growth, only 1214 genes were stage-specific to this transition (Figure 2). Similarly, 862 genes were stage-specifically upregulated in the transition from polar growth to elongation of the germ tube, and 2961 genes were stage-specifically upregulated during elongation to the first branching of the germlings. As germination progressed, a larger number of genes were upregulated than downregulated: 2961 genes were upregulated between elongation of the germ tube and first branching compared to 534 genes that were downregulated in this interval.

Based on their gene ontology (GO), 5313 of the genes that were well measured were attributed as components of biological processes, 2537 as cellular components, and 4633 as exhibiting molecular function. The majority of the genes expressed during germination were classified to transcription factor activity (411 genes), metabolic process (642 genes), and nucleic-acid binding (331 genes), all of which are apparently required for proper germination of this strain of *T. asperelloides*.

#### 3.2.1. Changes in Cellular Component Gene Expression Pattern during Conidial Germination

**Figure 3:**
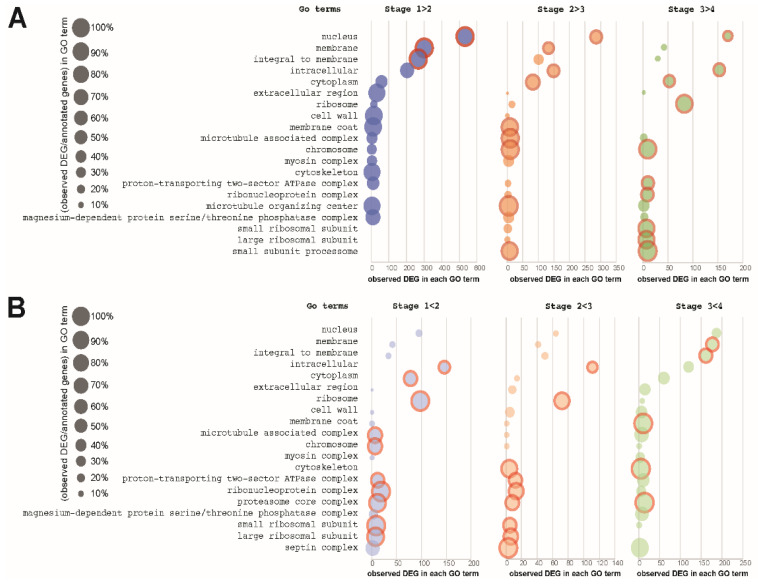
Selected 20 GO terms among the cellular components showing divergent enrichment patterns during phases of conidial germination of *T. asperelloides*. (**A**) Differentially expressed genes (DEGs) that were significantly downregulated during the process; (**B**) DEGs that were significantly upregulated during the process. Enrichment status is color-coded for different stage shifts. The bubble plot was scaled with the percentage of observed DEG for each GO term. Significantly enriched GO terms (Benjamini–Hochberg adjusted *p* < 0.05) are circled with red.

Genes belonging to the cellular-component functional group exhibited both high (“upregulated genes”) and low abundance of transcript (“downregulated genes”) throughout germination (Figure 3). Many functional categories—for example, nucleus-, cytoplasm-, and membrane-related genes—included genes that were both upregulated and downregulated in serial stages of germination. Some functions appeared to be stage-specific as transcript abundance was present at certain germination stages and was not detected in other stages (Table S3). Others, such as roles in the septin, proteasome core, and ribonucleoprotein complexes, appeared to be predominantly “upregulated” across stages of germination. In other cases—such as cytoskeletal genes—we determined that downregulation of some genes occurred from stage 1 to 2, and then upregulation occurred from stage 2 to 3 and from 3 to 4.

It was apparent that some cell-wall-related genes were “downregulated” from stage 1 to stage 2, yet others were “upregulated” throughout all stages of germination. Cell-wall-related genes and genes expressed to the outside of the plasma membrane were examples of functional subgroups that exhibited this pattern of expression, which implies the occurrence of de novo synthesis of the relevant components from the initial establishment of polarity through the entire germination process until first branching.

#### 3.2.2. Expression of Genes Associated with Cell Wall Remodeling during Germination

**Figure 4:**
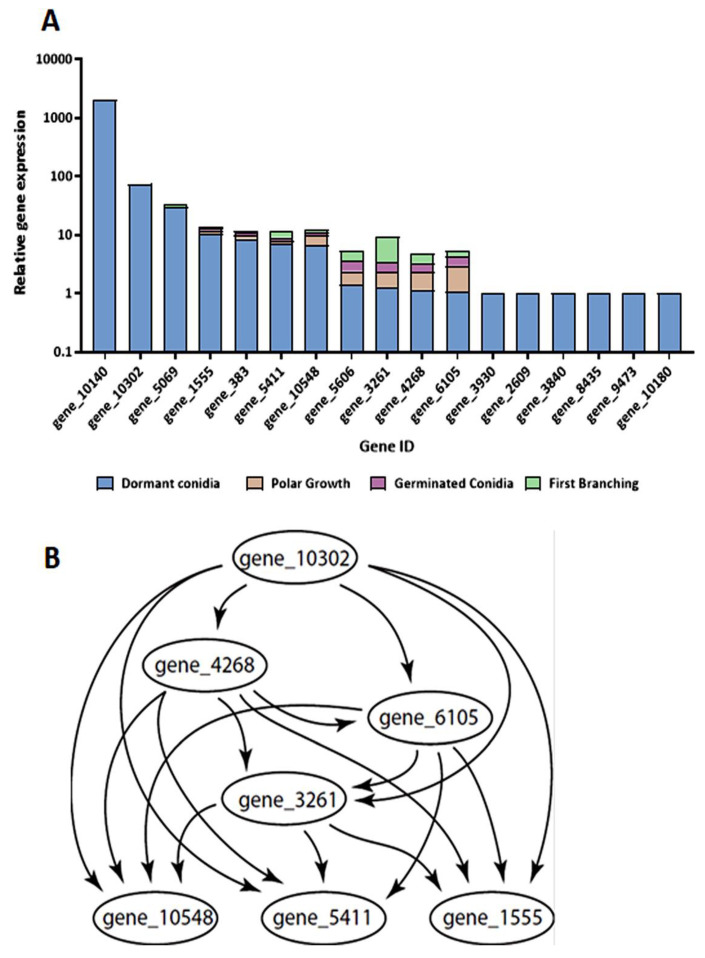
Relative gene expression and regulatory interactions of chitinases. (**A**) Log-scaled relative gene expression levels of chitinases across germination of *Trichoderma asperelloides*. Expression levels are expressed relative to the lowest (set as 1) among genes across the process. (**B**) Bayesian gene expression regulatory network was inferred from relative gene expression measurements for select chitinase-encoding genes.

Differential expression of genes associated with cell wall remodeling was found to occur during germination of *T. asperelloides*. Four gene families associated with cell wall remodeling and mycoparasitism were chosen as candidates for possible germination markers: chitinases and glucanases that participate in the degradation of the cell wall (self and host), as well as chitin synthases and glucan-elongases that function in the synthesis processes of cell wall remodeling. Among the 31 chitinase genes identified in the course of this study, only the transcripts of 17 (Tables S2 and S4) were detected during germination. Nine of these chitinases were differentially expressed, to different extents, throughout the germination process, and eight were classified as early genes that were detected only in dormant conidia (Figure 4). Gene 3261 appears to be the most prominent “late germination gene”; its transcript levels were highest following the onset of first branching. The presence of an extensive co-regulatory interaction between some chitinase-encoding genes was supported by Bayesian networks inferred from the transcriptional data, highlighting an upstream “core” of four chitinase genes (10,302, 4268, 6105, and 3261).

**Figure 5:**
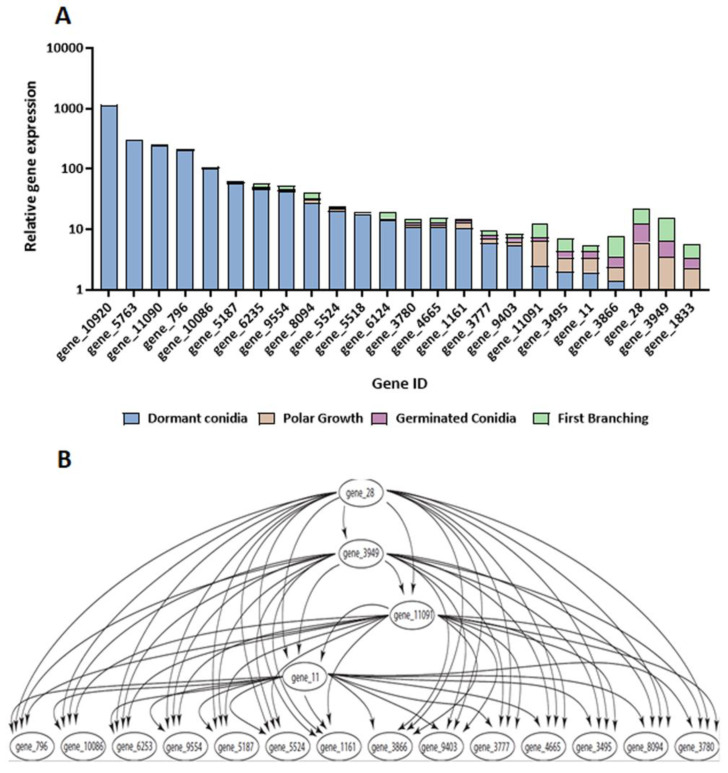
(**A**) Log-scaled relative expression levels of glucanases across conidial germination of *Trichoderma asperelloides*. Only glucanases with a minimal *r* value of 1 are presented. (**B**) Bayesian gene expression network, inferred from relative expression among select glucanase-encoding genes.

Similar to that observed in the case of the chitinases, we were able to detect the presence of about half of the glucanases along the timeline of germination (Figure 5A; Tables S2 and S4). Nonetheless, many of the glucanase transcripts were present mainly in dormant conidia, while three of them (28, 3949, and 1833) appeared to be preferentially transcribed from the onset of polar growth. Interestingly, the abundance of transcripts of “early genes” (those whose transcripts are predominantly detected in dormant conidia) can be, in many cases, approximately 10–100-fold higher than that found in genes that are expressed later in the developmental process. To examine co-regulation among glucanase-encoding genes, Bayesian networks were inferred from relative glucanase expression, highlighting a “core” of hub-regulatory glucanases composed of four genes (28, 3949, 11,091, and 11).

**Figure 6:**
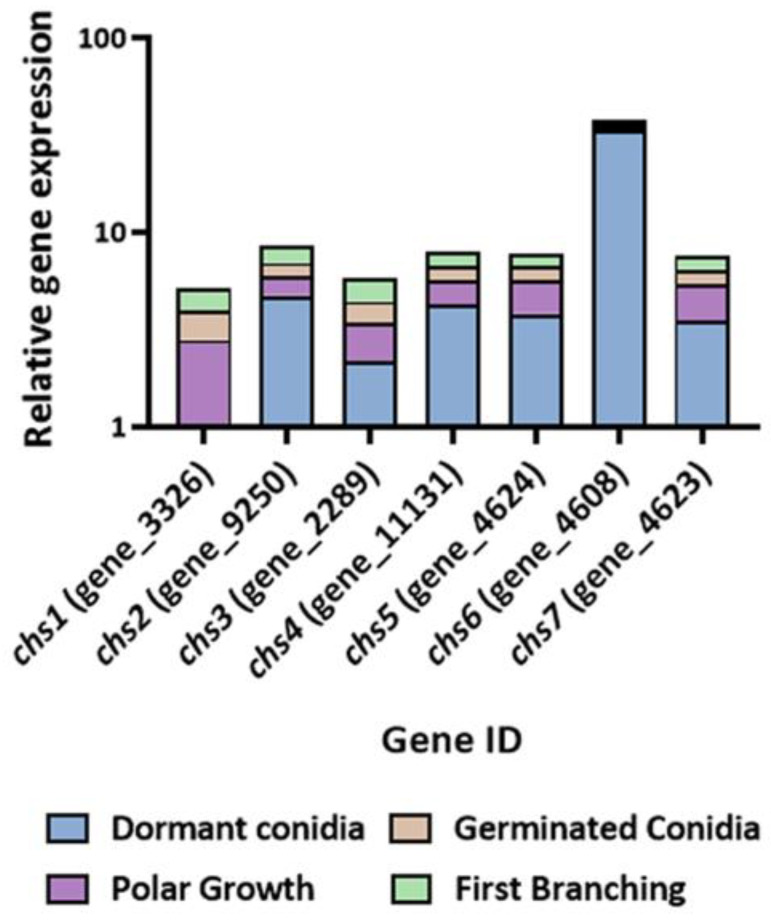
Log relative gene-expression levels of chitin synthases across the four-stage germination time course of *Trichoderma asperelloides*. Gene expression was determined by RT-PCR. Values shown are based on three biological repeats, with whiskers signifying one standard error. Gene expression was normalized to *tef1*. The threshold cycle (2^−ΔΔCT^) method was used to determine fold changes in expression.

**Figure 7:**
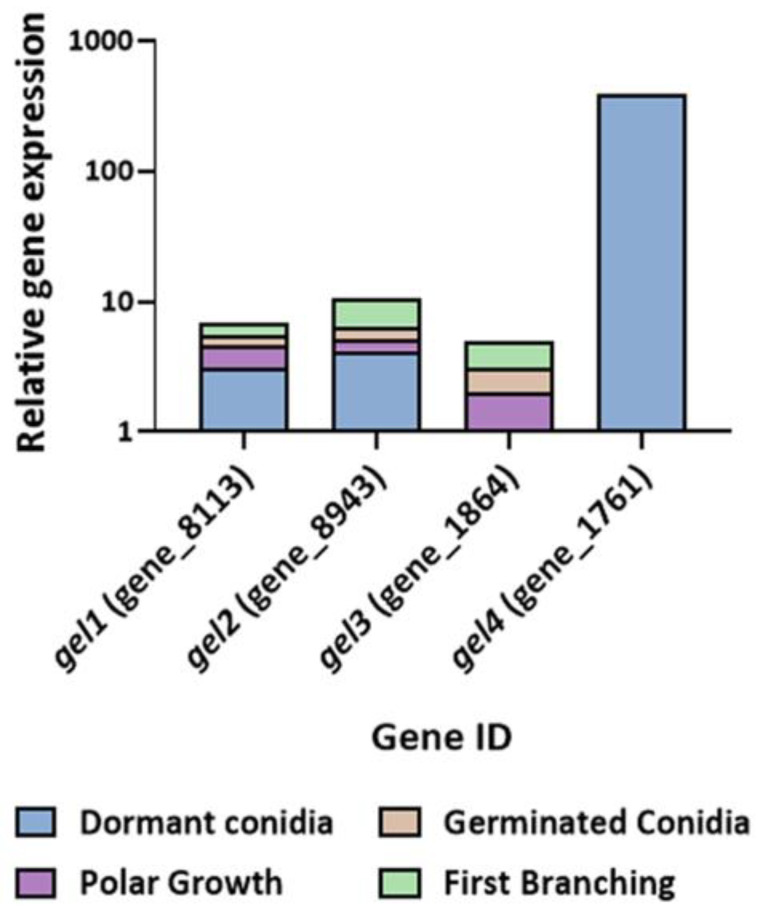
Log relative gene-expression levels of *Trichoderma asperelloides* glucan elongases across four stages of conidial germination. Gene expression was determined by RT-PCR. Values shown are based on three biological repeats, with whiskers signifying one standard error. Gene expression was normalized to *tef1*. The threshold cycle (2^−ΔΔCT^) method was used to determine fold changes in expression.

In addition to the chitinases and glucanases, we also compared the relative expression of genes encoding proteins that are involved in biosynthetic aspects of cell wall remodeling. These genes included chitin synthases and glucan elongases (Figure 6 and Figure 7). Seven of the eight *T. asperelloides* chitin synthases were differentially expressed during the four stages of germination (Figure 7; Tables S2 and S4). Transcripts of all the transcribed *chs* genes were detected in dormant conidia; *chs2* appeared to be the most abundantly present in that developmental phase. In contrast, *chs1* is an apparent “late” germination gene within the *chs* gene family, suggesting that it functions mainly after the onset of germination.

Four β-(1,3)-glucanosyltranferase genes were identified in the *T. asperelloides* genome. The members of this *gel* gene family were differentially expressed during germination, from dormancy to first branching. Transcripts of the genes *gel1* and *gel2* were most abundant in dormant conidia. However, they were also substantially transcribed along the time course of germination. Conversely, the *gel3* gene was highly expressed “late”: even though its transcripts were present in conidia, they were the most abundant compared to the other *gel* genes in the other phases of germination (Figure 7).

### 3.3. Congo Red Affects Germination and Alters Expression of gel3

**Figure 8:**
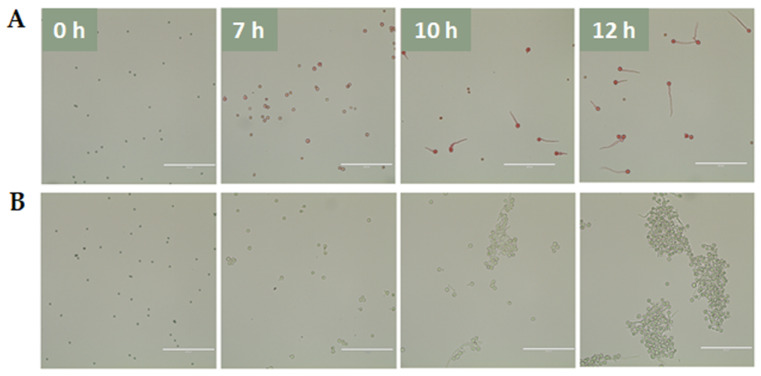
Germination of T203 conidia imaged at four time points (white numbers). Germination in (**A**) 0.5 mM Congo Red and (**B**) PDB. Scale bar: 100 µm for all images.

**Figure 9:**
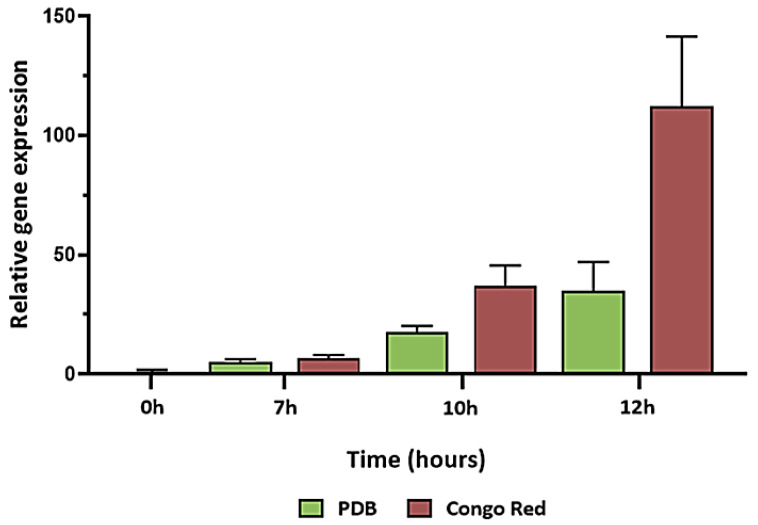
Expression levels calculated as of *gel3* during germination in PDB and in the presence of 500 µM Congo Red. Gene expression was determined by RT-PCR. Values shown are based on three biological repeats, with whiskers signifying one standard error. Gene expression was normalized to *tef1*. The threshold cycle (2^−ΔΔCT^) method was used to determine fold changes in expression. Standard errors (whiskers) were based on three biological replicates.

Given the changes occurring in the transcript abundance of *gel3* during germination and the documented significance of GEL3 homologues in cell wall integrity [35,36], we monitored *gel3* expression during germination in liquid medium (PDB) cultures challenged with a sub-lethal concentration (500 µM) of Congo Red. While initiation of polarity did not seem to be affected by the presence of the dye (Figure 8, 7 h), only about 50% of the conidia progressed along the germination timeline beyond that initial stage. Those conidia that did germinate produced normal germ-tubes. However, they failed to branch within the time course of this experiment. Moreover, the characteristic branching and clumping observed in control culture germlings was not evident in the treated cultures. These phenotypic effects of Congo Red on germling development were accompanied by increases in the abundance of *gel3* transcripts that were most evident at later stages of germination. After 10 h of incubation, the expression of *gel3* was two-fold higher in the treated conidia. The difference increased to five-fold after 12 h of incubation with the dye (Figure 9). These gene expression effects of the inhibitor further support the presence of phase-specific checkpoints during germination and the ability of the developing germlings to respond to cell wall remodeling insults by altering gene expression of key components of this process.

### 3.4. Changes in Expression of Chitin-Synthase- and Glucan-Elongase-Encoding Genes in the Presence of Rhizoctonia solani

**Figure 10:**
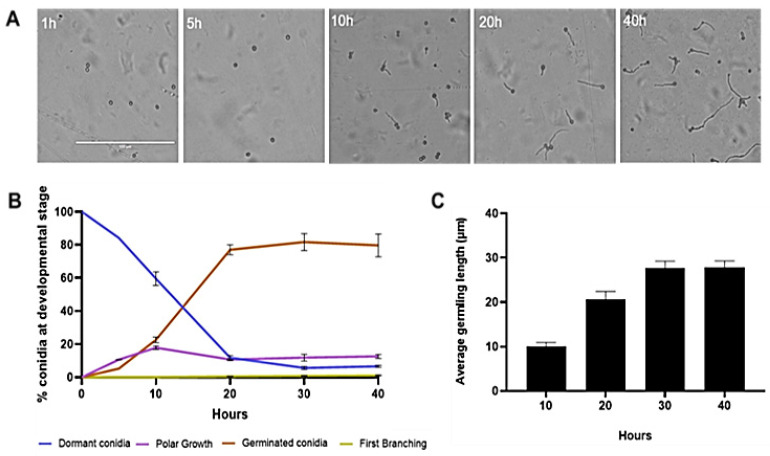
Phenotypic characterization of conidia germinating on *R. solani* host. (**A**) Conidia germinated on *R. solani* were imaged at different time points, as indicated. Scale bar: 100 µm for all images. (**B**) Conidial germination as a percentage over time, determined by live-cell imaging. (**C**) Average germling length, quantified by *n* = 50 randomly selected conidia for each time point. Error bars describe standard deviations of the mean for three biological replicates.

Genes of the *chs* and *gel* families were differentially expressed during germination on PDA. Therefore, we examined to what extent the expression pattern of these genes was dependent on the germination substrate. We determined the level of expression of these genes in the presence of a potential host. First, we observed the morphological dynamics of conidial germination of *T. asperelloides* conidia plated on membranes that had been placed on a live culture of *R. solani*. In contrast to the transition from dormancy to swelling that was observed during germination on PDA, the transition from dormancy to swelling on live culture was very subtle and challenging to quantitate. The emergence of germ tubes from the conidia was observed after ten hours, and elongation of the germ tube proceeded for about 40 h, with the average germling length reaching 25 µm after 30 h (Figure 10). Most conidia (75–80%) took almost twice as long (20 h) to germinate as what was measured on PDA. Based on these observations, and on the observation that *T. asperelloides* was able to germinate at normal rates even on water agar (data not shown), we concluded that the marked delay, as well as alteration in the synchronized progression of the germination program, was most likely linked to the presence of *R. solani*.

**Figure 11:**
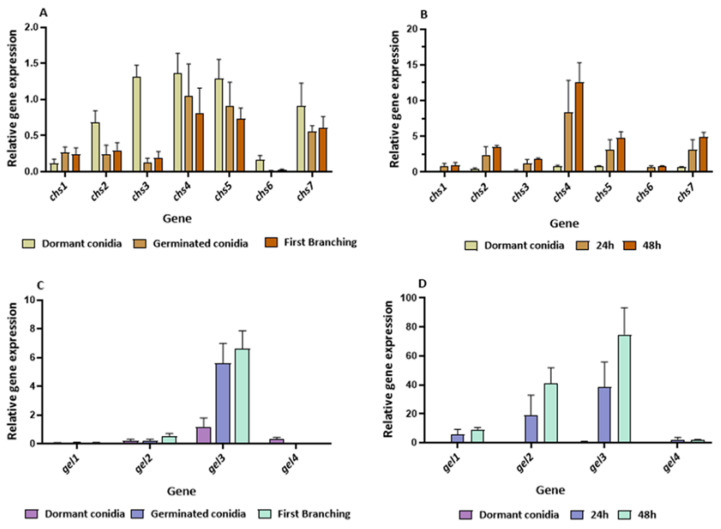
Expression levels of *chs1*–*chs7* during germination (**A**) on solid medium or (**B**) on the host *Rhizoctonia solani.* Expression levels of *gel1*–*gel4* genes during germination (**C**) on solid medium or (**D**) on the host *R. solani*. Gene expression was determined by RT-PCR. Values shown are based on three biological repeats, with whiskers signifying one standard error. Gene expression was normalized to *tef1*. The threshold cycle (2^−ΔΔCT^) method was used to determine fold changes in expression.

The difference in the germination timeline was accompanied by marked changes in the transcript abundance of genes in the *chs* and *gel* families, as determined by RT-PCR (Figure 11). While the expression patterns of the *chs* gene family on PDA, as determined here, were similar to those determined by RNASeq, the relative expression of members of the *chs* gene family was ten-fold greater in conidia that germinated on the host *R. solani* when compared to the expression measured on PDA (Figure 11A,B). In addition to the increase in *chs* transcript abundance, the pattern of the *chs* transcript during germination on the two substrates also varied. In fact, the expression pattern of the *chs* gene family in the presence of a host appeared as a “mirror image” of the expression patterns under conditions representing a saprotrophic lifestyle of the mycoparasite (on PDA). Most chitin synthase genes, excluding *chs1* and *chs6*, were highly expressed during germination on the host and shared a similar expression pattern. 

Among the four *gel* genes, *gel3* was the most highly expressed in both germinating conidia and after the onset of the first hyphal branching in cultures growing on PDA. As in the case of the *chs* transcripts, the relative expression of members of the glucan-elongase family was ten-fold greater in conidia that germinated on the host *R. solani* when compared to their expression on PDA (Figure 11C,D). Excluding *gel4*, all the *gel* genes were upregulated during germination in the presence of *R. solani.* Among them, *gel3* was the most highly expressed in the germinating conidia.

Based on these results, we concluded that, even though most of the cell wall remodeling gene transcripts are present in dormant conidia, the dynamics of their transcriptional abundance can be significantly altered during the germination process in a manner that is dependent on the presence of this potential host. 

## 4. Discussion

Here, we have shown that a functional classification of differentially expressed transcripts of germinating *T. asperelloides* conidia is characterized by an increase in various metabolic processes and electron transport, as well as enhancements in transcription factor activity, nuclear activity, nucleic-acid binding activity, and protein-kinase activity, all accompanying a striking and morphologically dynamic developmental process. The transcriptional profile of germinating *T. asperelloides* featured 11,166 genes whose transcript abundance changed during the fungus germination process. About half (5550 genes) were uniquely downregulated in the transition from dormancy to polar growth, and about a quarter (2961) were exclusively upregulated in the transition from elongation of the germ tube to first branching.

This dynamism of expression indicates the presence of an abundant repertoire of mRNA species that are likely to be produced during conidiogenesis. This was followed by the utilization of the pre-existing RNAs for the onset of germination and subsequent de novo synthesis of RNAs that were used in more advanced stages of germination. RNAs have been shown to be synthesized in the “dormant” conidia of *A. nidulans*, *A. fumigatus,* and *Talaromyces marneffei* [6]. Therefore, it is likely that such processes occur prior to germination in *Trichoderma* spp. as well. As opposed to loading of RNAs into forming conidia, the extent to which RNA synthesis within dormant conidia contributes to the extremely high representation of detectable RNA species (10,725, comprising over 95% of the predicted gene number in this species) has yet to be determined. However, the possible physiological/ecological implications of such a comprehensive diversity of transcripts are vast.

A similar, albeit less pronounced, trend in transcript abundance was observed in germinating conidia of *N. crassa* conidia [7]. There, transcripts of about a third of the *N. crassa* genome were present in the dormant conidium, and about 14% were exclusively upregulated during first branch formation. When comparing the expression patterns between the stages of germination in *N. crassa*, it was observed that most of the transcripts present in the dormant conidia were absent at later stages of germination and that 40 times as many genes were downregulated in dormant conidia compared to germinated conidia [7]. In the example of *A. niger*, transcripts of 33% of the annotated genes were present in the dormant conidia, and the same fraction was detected in germinating conidia [10]. In *Rhizopus delemar*, transcripts representing over 70% of the genome were identified in resting spores. Only a slight decrease in the percentile of the genome represented was evident once germination had initiated. However, a shift in the abundance of different functional groups was observed [55]. Some of the transcripts that were unique to the resting spores of *R. delemar* included those that have predicted roles in lipid storage and localization and were suggested to be involved in dormant spore maintenance.

Interestingly, we were also able to identify several such lipid-related gene transcripts in dormant conidia of *T. asperelloides*. Among them were plasma-membrane proteolipid 3 and cyclopropane-fatty-acyl-phospholipid synthase (gene IDs 2212 and 8825, respectively; Table S2), whose transcript levels dropped to less than 0.1% once germination was initiated. In contrast, transcriptional profiling of germinating *F. graminearum* conidia showed similarity in both the number and regulation of genes expressed throughout different stages of germination. Of the 13,969 genes annotated, 1805 genes were upregulated and 1616 genes were downregulated in dormant conidia, while, in germinated conidia, 954 and 947 genes were upregulated and downregulated, respectively [12]. Taken together, it is clear that dynamic changes in transcript abundance of a substantial portion of the coding genome is a hallmark of fungal conidial germination. However, the relative proportions of the coding genomes involved vary among fungal species. In *T. asperelloides*, the changes we observed were among the most dynamic described to date in terms of the percentage of the genome involved. Members of the genus *Trichoderma* can be found in almost every conceivable niche in nature, and, in addition to their saprophytic nature, many species can be associated with a multitude of living fungi and plant hosts [14,17,20]. Therefore, these fungi are likely to have evolved to produce conidia with a repertoire of available transcripts that provide the metabolic preparation to germinate and thrive on diverse substrates.

Cell wall remodeling is a critical process involving many genes in various functional groups during germination. Changes in the abundance of transcripts encoding proteins involved in cell wall remodeling were especially striking. The abundances of many of these transcripts were reduced during the early germination (stage 1 < stage 2) but were synthesized de novo during late germination (stage 3 < stage 4). Cell wall remodeling includes both the biosynthesis as well as the degradation of cell wall components [9]. Degradation of the rigid conidial cell wall is a prerequisite for progression of the germination process and may depend on the activities of a range of hydrolytic enzymes. Several genes accounting for these enzymes were identified in transcriptomic and proteomic studies during the germination of *Aspergillus* sp. [9,11,56,57]. Among them, there were genes for several chitin synthases and 1,3-β-glucanosyltransferases. Regulatory associations among genes in the functional groups of interest were reconstructed via Bayesian gene networks using expression fold-change collected from the four major morphological stages in conidial germination in *T. asperelloides*. Such networks provide preliminary working hypotheses that can productively guide future gene manipulation experiments that efficiently and rigorously assess regulatory networks for these functional groups. Here, Bayesian networks of the chitinases and glucanases identified a selection of genes that comprise a core of chitinases and glucanases featuring the most marked stage-specific expression changes during germination. We identified several potential “core” genes with marked changes observed in expression. These core genes may serve as functional stage-specific markers for further elucidation of the genetics and regulation of conidial germination in this species and its close (if not distant) relatives.

For efficiency of resource use, the degradation of existing cell wall material must be coordinately orchestrated with de novo synthesis of the cell wall material in the developing germling. Much attention has been devoted to the involvement of chitin synthases in cell wall biosynthesis in a variety of fungi [31,56,58,59,60,61]. For example, the differential expression of the eight-member *T. atroviride* chitin synthase gene family during germination and vegetative growth has been characterized [33]. However, under the conditions tested here, the transcripts of only seven of the eight *T. asperelloides* chitin synthases were above the conventional threshold for detection (Figure 6). The detectable transcript abundance of all *chs* genes was highest in dormant conidia, with the exception of *chs1*, whose transcript was present only from stage 2 of germination. While the *N. crassa* homologue of this gene, *chs-2*, was found to be non-essential [62,63], it was found to localize to the Spitzenkörper, which would certainly be expected in a germling, especially once polarity has been established. In contrast, while we found that *chs7* transcript levels were highly abundant in conidia, the mRNAs of this gene were almost undetectable once germination had been initiated but were found at high levels in mature hyphae [64]. As mentioned, the transcript of *chs8* was below detection levels under the conditions tested. The transcript levels of this gene were reported as being very low during vegetative development yet highly abundant when the fungus was exposed to osmotic or oxidative stress [64].

Another major polymer in the fungal cell wall is β-1,3-glucan, which can be remodeled by members of the GH72 of the glycoside hydrolase family of proteins, also known as glucan elongases (GELs) [34,35]. Most fungi have several GEL-encoding genes, and the expression of each gene may be differentially regulated during various growth stages and/or environmental conditions. *N. crassa* has five *gel* genes, among which *gel-3* was most strongly expressed both in germinating conidia and during hyphelongation. Deletion of *gel-3* affected vegetative growth, causing slow growth and abnormal morphology [65]. To date, 1,3-β-glucanosyltransferases have not been studied in *Trichoderma*. In this study, the expression of four GEL-encoding genes was detected along the germination timeline. At the same time, *gel1–3* exhibited lower, albeit differential, expression during all stages of germination. Among these three *gel* genes, that of *gel3* appeared to be lowest in the dormant conidia, with higher levels of expression observed after the onset of conidiation. To determine whether *gel3* expression is not only dependent on the developmental program of germination but could also be induced following external cell-wall-related aggravation, we measured *gel3* transcript abundance in cultures challenged with cell wall perturbing agent Congo Red [66]. During the early phases of germination, the effect of Congo Red on the *gel3* transcript levels was minor. However, after 12 h of incubation with the dye *gel3,* the transcript levels were almost five-fold higher when compared to the control. One possible explanation for the late increase in transcript abundance is that the intact thicker conidium cell wall acts as an effective physical barrier against the dye during the pre-germling phase. Alternatively, it is possible that the drug must be taken up into the cell and processed to be effective and that the machinery involved is expressed only at later stages of germination. Gene expression analysis during exposure of growing mycelial cultures of *A. fumigatus* to Congo Red revealed overexpression of Gel4 and Gel7, suggesting that the presence of Congo Red activates the reorganization of β-1,3-glucans through processing enzymes [67].

To date, the mycoparasitism of *T. asperelloides* strain T203, as well as other *Trichoderma* spp. strains, has been extensively studied in advanced developmental stages, mainly during hyphal coiling around its hosts [68,69]. This study has focused on the processes underlying early developmental and pre-infection stages. Germination of *T. asperelloides* was morphologically and temporally distinct when cultured on PDA versus when cultured in the presence of the host *R. solani* (Figure 1 and Figure 10). Germination in the presence of *R. solani* was ten-fold slower, and the germlings appeared shrunken. Furthermore, they elongated for extended periods of time (ten to twenty hours) prior to branching. The germination pattern observed could be attributable to the potential host, whose structure or exudates affect the germination process. In concert with slower germination, the transcript abundance of several genes involved in cell wall remodeling was significantly (up to 10-fold) higher. These changes in transcript abundance are in agreement with recent research demonstrating a prey-sensing, recognition, and directional chemotropic approach during the early phases of interaction [70,71]. Therefore, the high transcript levels observed may be related to the mentioned processes. Interestingly, the highest levels of *chs* gene expression in the presence of the host were observed in the case of *chs4, chs5,* and *chs7*, especially at the later stages of germination. Homologues of *chs4*, *chs5,* and *chs7* in *T. atroviridae* were highly expressed during mycoparasitism [64]. Our data support the presence of a transcriptional response to the presence of a host during germination. However, the presence of such a response does not rule out the possibility that slower mRNA turnover could also contribute to the higher transcript levels observed.

In this study, we have demonstrated that conidial germination in a member of the highly ubiquitous genus *Trichoderma* is among the most dynamic, in terms of changes in transcript abundance, compared to other species analyzed to date. The expression changes observed here are likely accompanied by many additional changes in the biochemical and physiological state of the germinating fungus, as has been shown in other species [72,73,74]. The extent to which transcriptional hallmarks of germination are conserved under varying conditions in diverse fungi has yet to be determined. Comparative transcriptional analyses are likely to provide both evolutionary insights concerning fungal development and lifestyles, as well as means for identifying and eventually manipulating key regulatory elements in this fundamental process.

## Figures and Tables

**Table 1:** Primers used in this study.

Gene Name	Primer Designation	Sequence (5′ to 3′)
Translation elongation factor 1α	tef1α F tef1α R	TGACGGCAACCCCACTATCG CGAGTTCGGCGGCTTCCTAT
Chitin synthase 1	CHS1F CHS1R	CATTACGGTGTTGCCCGGTG GCCCATGGCCAGTTTCATCG
Chitin synthase 2	CHS2F CHS2R	CCGACGGGTGGCATCAAAAG GTTCGCCGTGAGGGTTTTCG
Chitin synthase 3	CHS3F CHS3R	CGCCAAGCAGCAAGTGAACA GTGTTCGGCGATGAACCAGC
Chitin synthase 4	CHS4F CHS4R	GGGTATTCCGTTGGAGGCGA GAGCCCTCAGCAAGGGTGAT
Chitin synthase 5	CHS5F CHS5R	GATACCGTTGTCGCCCCAGA ATTAGTCAGGGCCGTCTCGC
Chitin synthase 6	CHS6F CHS6R	ACCGCCATCGTTGGTGTAGT CACGCAGAGCACGTTGATGG
Chitin synthase 7	CHS7F CHS7R	CACCGTACTCCGACTTCCCC ACCGAAGCCTGGGGATAAGC
Glycosyl-elongase 1	GEL1F GEL1R	GTGAGATCCCCGTCGGCTAC GGGTCGCACCACGAATAGGA
Glycosyl-elongase 2	GEL2F GEL2R	GAACCGACTTTGTCGACGCC CCCGACTTGGGGTCGTATCC
Glycosyl-elongase 3	GEL3F GEL3R	ACGACGTCGATATCCGCTCC AGCGGTCTCGGTGTCATAGC
Glycosyl-elongase 4	GEL4F GEL4R	ACTTTGCCGCGCTTCAGAAC ACTGCTGGGCAGTCTTCAGG

## Supplementary Materials

The following supporting information can be downloaded at: https://www.mdpi.com/article/10.3390/jof8080854/s1, Table S1: RNAseq statistics for 4 stages of conidial germination of *T. asperelloides* on PDA.; Table S2: LOX measurements of *T. asperelloides* transcriptomics with detailed functional annotations.; Table S3: GO terms enrichment analysis during *T. asperelloides* condial germination. Genes that are significantly differentially expressed were annotated for each pair of stage comparisons; Table S4: Gene designations and links for Chitinases, Glucanases, Chitin Synthases and Glucan Elongases.
